# The Nucleocapsid Proteins of SARS-CoV-2 and Its Close Relative Bat Coronavirus RaTG13 Are Capable of Inhibiting PKR- and RNase L-Mediated Antiviral Pathways

**DOI:** 10.1128/spectrum.00994-23

**Published:** 2023-05-08

**Authors:** Kyle LeBlanc, Jessie Lynch, Christine Layne, Robert Vendramelli, Angela Sloan, Nikesh Tailor, Yvon Deschambault, Fushun Zhang, Darwyn Kobasa, David Safronetz, Yan Xiang, Jingxin Cao

**Affiliations:** a Poxviruses and Vaccine Design, Division of Viral Diseases, Directorate of Science Reference and Surveillance, National Microbiology Laboratory, Public Health Agency of Canada, Winnipeg, Manitoba, Canada; b Special Pathogens, Division of Health Security and Response, Directorate of Scientific Operations and Response, National Microbiology Laboratory, Public Health Agency of Canada, Winnipeg, Manitoba, Canada; c Department of Microbiology and Immunology, The University of Texas Health Science Center, San Antonio, Texas, USA; Institute of Molecular Biology, Academia Sinica

**Keywords:** G3BP1, PKR, RNase L, RaTG13, SARS-CoV-2 nucleocapsid, double-stranded RNA virus, vaccinia virus E3

## Abstract

Coronaviruses (CoVs), including severe acute respiratory syndrome CoV (SARS-CoV), Middle East respiratory syndrome CoV (MERS-CoV), and SARS-CoV-2, produce double-stranded RNA (dsRNA) that activates antiviral pathways such as PKR and OAS/RNase L. To successfully replicate in hosts, viruses must evade such antiviral pathways. Currently, the mechanism of how SARS-CoV-2 antagonizes dsRNA-activated antiviral pathways is unknown. In this study, we demonstrate that the SARS-CoV-2 nucleocapsid (N) protein, the most abundant viral structural protein, is capable of binding to dsRNA and phosphorylated PKR, inhibiting both the PKR and OAS/RNase L pathways. The N protein of the bat coronavirus (bat-CoV) RaTG13, the closest relative of SARS-CoV-2, has a similar ability to inhibit the human PKR and RNase L antiviral pathways. Via mutagenic analysis, we found that the C-terminal domain (CTD) of the N protein is sufficient for binding dsRNA and inhibiting RNase L activity. Interestingly, while the CTD is also sufficient for binding phosphorylated PKR, the inhibition of PKR antiviral activity requires not only the CTD but also the central linker region (LKR). Thus, our findings demonstrate that the SARS-CoV-2 N protein is capable of antagonizing the two critical antiviral pathways activated by viral dsRNA and that its inhibition of PKR activities requires more than dsRNA binding mediated by the CTD.

**IMPORTANCE** The high transmissibility of SARS-CoV-2 is an important viral factor defining the coronavirus disease 2019 (COVID-19) pandemic. To transmit efficiently, SARS-CoV-2 must be capable of disarming the innate immune response of its host efficiently. Here, we describe that the nucleocapsid protein of SARS-CoV-2 is capable of inhibiting two critical innate antiviral pathways, PKR and OAS/RNase L. Moreover, the counterpart of the closest animal coronavirus relative of SARS-CoV-2, bat-CoV RaTG13, can also inhibit human PKR and OAS/RNase L antiviral activities. Thus, the importance of our discovery for understanding the COVID-19 pandemic is 2-fold. First, the ability of SARS-CoV-2 N to inhibit innate antiviral activity is likely a factor contributing to the transmissibility and pathogenicity of the virus. Second, the bat relative of SARS-CoV-2 has the capacity to inhibit human innate immunity, which thus likely contributed to the establishment of infection in humans. The findings described in this study are valuable for developing novel antivirals and vaccines.

## INTRODUCTION

Severe acute respiratory syndrome coronavirus 2 (SARS-CoV-2) is the causative agent of the coronavirus disease 2019 (COVID-19) pandemic (1). Currently, there are seven coronaviruses causing diseases in humans: four cause generally mild respiratory diseases (229E, NL63, OC43, and HKU1), while three are capable of causing more severe respiratory diseases (SARS-CoV, SARS-CoV-2, and Middle East respiratory syndrome coronavirus [MERS-CoV]) (2, 3). Although the origin of SARS-CoV-2 is still unclear, it is likely that the virus crossed the species barrier to infect humans from an animal origin, similar to other human coronaviruses (4, 5). SARS-CoV-2 infection in humans is highly transmissible, with a wide range of severities ranging from asymptomatic to lethal. SARS-CoV-2 is a betacoronavirus with a single-stranded, positive-sense, and ~30-kb RNA genome that encodes 4 structural proteins (spike [S], membrane [M], envelope [E], and nucleocapsid [N]), 16 nonstructural proteins (NSP1 to -16), and 9 accessory proteins (1).

Innate immunity provides the first line of defense against virus infection. To establish a productive infection, a virus must be capable of evading the host’s innate immunity. During the replication cycle of a virus, the virus produces pathogen-associated molecular patterns (PAMPs), such as double-stranded RNA (dsRNA), which are detected by the pattern recognition receptors (PPRs) (e.g., RIG-I/MDA5 for dsRNA) of the host (6). The interaction between a PRR and a PAMP, such as RIG-I/MDA5 and dsRNA, activates a signaling pathway cascade that leads to the production and secretion of interferon (IFN) and other cytokines/chemokines. Type I interferons are a cornerstone of innate immunity against virus infections. IFNs bind to their receptors and activate the transcription of hundreds of interferon-stimulated genes (ISGs), many of which encode proteins with antiviral activities. PKR and OAS/RNase L are important antiviral proteins that can be induced by type I IFNs and directly activated by dsRNA. Once activated, PKR phosphorylates the α subunit of eukaryotic initiation factor 2 (eIF2α), resulting in the arrest of protein translation, while RNase L degrades RNAs, including viral mRNAs (7, 8).

Coronaviruses produce dsRNA during their replication, which can activate antiviral proteins such as PKR and RNase L (9–11). SARS-CoV-2 has been shown to activate both PKR and RNase L in a cell type-dependent manner (12). Interestingly, activated PKR and RNase L pathways do not seem to inhibit SARS-CoV-2 replication, indicating that SARS-CoV-2 is capable of inhibiting the antiviral activities mediated by PKR and RNase L (12). Using an ectopic-expression system, it was reported previously that SARS-CoV-2 N can interact with G3BP1 and PKR, and such an interaction is associated with the inhibition of stress granule (SG) formation (13–15). However, it is still not clear how such interactions between the N protein and G3BP1/PKR may contribute to the inhibition of the antiviral activity mediated by G3BP1/PKR. In addition, it is not known if SARS-CoV-2 N is capable of inhibiting RNase L-mediated antiviral activity.

Vaccinia virus (VACV), the prototype poxvirus, encodes two proteins, E3 and K3, to suppress dsRNA-activated PKR and/or RNase L antiviral pathways. E3, a dsRNA binding protein, inhibits both PKR and RNase L, while K3 (eIF2α homolog) modulates PKR antiviral activity by interfering with the phosphorylation of the PKR substrate eIF2α (16, 17). Previously, we have shown that the deletion of the VACV E3L and K3L genes (VACVΔE3ΔK3) renders the virus unable to replicate in cells with functional PKR and/or RNase L pathways (18; Jingxin Cao, unpublished data). Thus, a protein with the suspected ability to suppress PKR and/or RNase L antiviral activities can be examined using this VACVΔE3ΔK3L platform, and the restoration of virus replication in cells with functional PKR and/or RNase L activities would demonstrate the inhibition of PKR and/or RNase L activity by that protein.

In this investigation, we expressed the SARS-CoV-2 N protein in VACVΔE3ΔK3 and demonstrated that the SARS-CoV-2 N protein can restore virus replication in cells by suppressing both PKR and RNase L antiviral activities. Furthermore, we identified the functional domains of the N protein that are essential for suppressing PKR and RNase L. Finally, we found that the N protein of the bat coronavirus (bat-CoV) RaTG13, the closest relative of SARS-CoV-2, is also capable of inhibiting human PKR and RNase L, indicating that the bat-CoV has the evasion measures to counteract human innate immunity.

## RESULTS

### SARS-CoV-2 suppresses dsRNA-activated antiviral activity.

**FIG 1:**
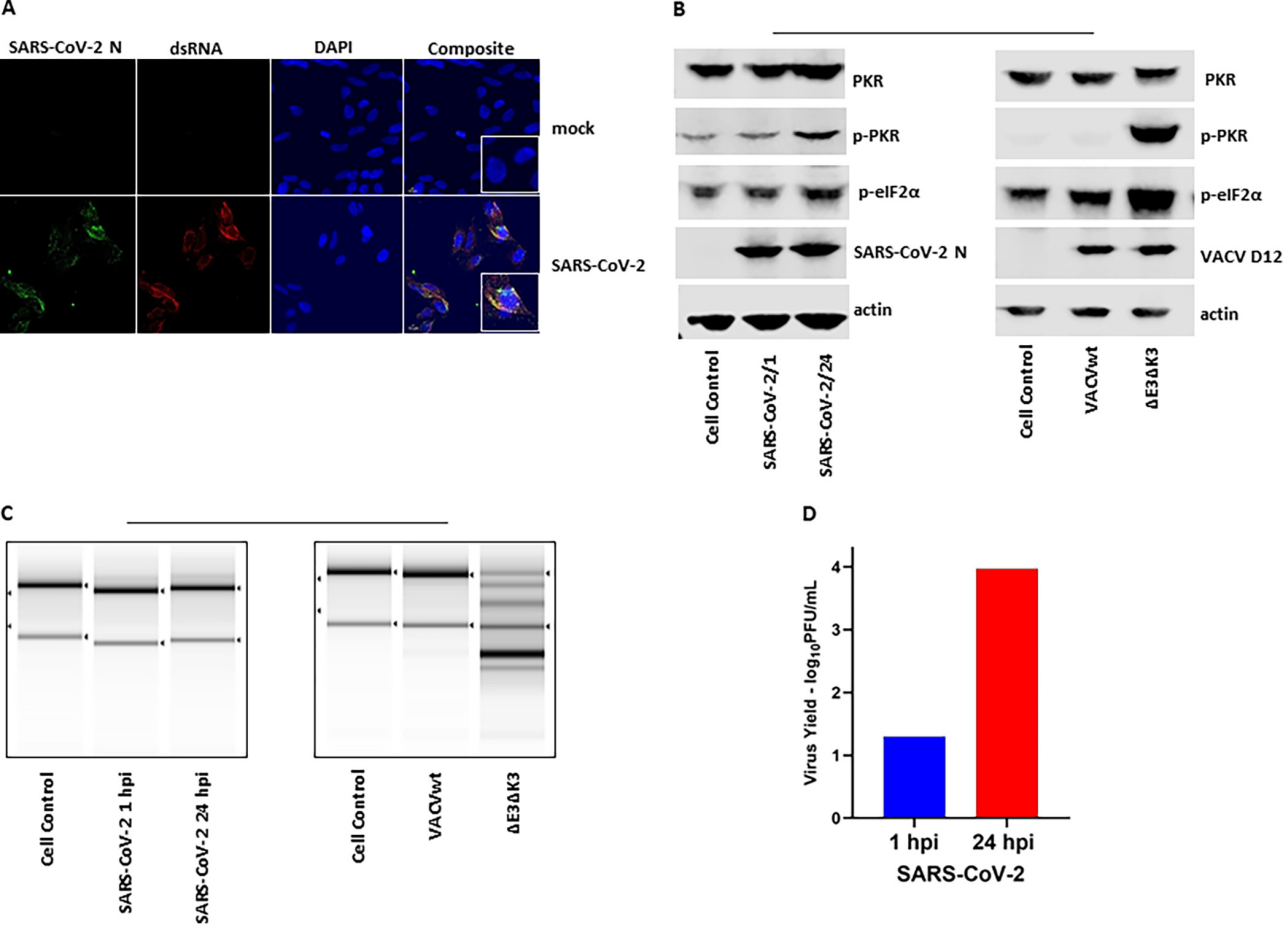
SARS-CoV-2 replication produces dsRNA and does not activate PKR and OAS/RNase L pathways. (A) Immunostaining of dsRNA in SARS-CoV-infected A549/huACE2 cells. Cells were infected with SARS-CoV-2 at an MOI of 0.5, incubated for 24 h, fixed with PBS-buffered formalin, and stained with a dsRNA-specific antibody (J2), antibody for the N protein, and DAPI for the nucleus. The images were taken at a ×40 magnification. The data are representative of results from two independent experiments. (B) Status of PKR and eIF2α phosphorylation. A549/huACE2 cells were infected with SARS-COV-2 at an MOI of 0.5 or VACVwt/VACVΔE3ΔK3 at an MOI of 5. Cell lysates were collected at 24 hpi for SARS-CoV-2-infected cells and 10 hpi for VACV-infected cells. SARS-CoV-2 N and VACV D12 Abs were used as controls for infection. The data are representative of results from three independent experiments. (C) RNA electrophoresis. Cells were infected as described above for panel B. The total RNA was collected for electrophoresis. The integrity of the rRNA is shown as an indicator of RNA degradation. The data are representative of results from two independent experiments. (D) SARS-CoV-2 yield. A549/huACE2 cells were infected with SARS-CoV-2 at an MOI of 0.5, and the supernatant was collected at 1 and 24 hpi for quantification of the virus by plaque assays on Vero cells. The data are representative of results from two independent experiments.

A previous study reported that SARS-CoV-2 infection leads to the activation of PKR and RNase L in A549 cells expressing human ACE2 (A549/huACE2) at 48 h postinfection (hpi). However, virus replication was not affected at either 24 or 48 h postinfection (12). It is not clear if the unaffected virus replication was due to the lack of dsRNA and/or activated PKR/RNase L at an earlier time point after infection. Here, we examined dsRNA production, the activation of PKR, and virus replication in A549/huACE2 cells at 24 h postinfection. The presence of dsRNA was examined using an immunofluorescence assay (IFA) with a J2 monoclonal antibody (Ab) that specifically recognizes dsRNA species of >40 bp in length (9, 19). As shown in Fig. 1A, dsRNA was detected extensively 24 h after SARS-CoV-2 infection, while no dsRNA was observed in uninfected cells. Interestingly, the N protein colocalized with dsRNA. To investigate if the dsRNA produced during SARS-CoV-2 replication activated PKR, the cell lysate collected at 24 hpi was examined for PKR phosphorylation at Thr446. As shown in Fig. 1B, only a trace amount of phosphorylated PKR (p-PKR) was detected in the SARS-CoV-2-infected cells. To confirm if the PKR- and OAS/RNase L-mediated antiviral pathways are functional in the cells, PKR in the cells infected with VACV with its two PKR inhibitor proteins deleted (VACVΔE3ΔK3) was almost completely phosphorylated, as indicated by the reduced mobility of the PKR protein in the cells infected by the mutant VACV. Similarly, the OAS/RNase L activity was confirmed by examining the degradation of 28S/18S rRNA following virus infection. No degradation of rRNA was observed in the SARS-CoV-2-infected cells at 24 hpi (Fig. 1C). In contrast, the majority of the rRNA was cleaved in the cells infected with VACVΔE3ΔK3. The replication of SARS-CoV-2 was examined by comparing the virus titers at 1 hpi and 24 hpi, and we found that the virus titers were increased by more than 100-fold at 24 hpi (Fig. 1D). Thus, SARS-CoV-2 replication produces a significant level of dsRNA, but it does not activate PKR and OAS/RNase L. It is known that early coronavirus replication occurs within the endoplasmic reticulum-associated double-membrane vesicles (20). It is likely that the double-membrane vesicles may contribute to shield the dsRNA produced during early replication from being detected by host pattern recognition receptors such as PKR and OAS/RNase L. However, it was previously reported that the ns2 protein of mouse hepatitis virus (MHV) plays a role in inhibiting OAS/RNase L activity as early as 9 h postinfection (11). Therefore, our observations indicated that SARS-CoV-2 might have another mechanism, in addition to the double-membrane-associated vesicles, to suppress dsRNA-activated antiviral activities to facilitate its replication.

### SARS-CoV-2 N protein binds dsRNA and inhibits PKR/RNase L activities.

As shown in Fig. 1A, the SARS-CoV-2 N protein colocalized with dsRNA in the infected cells. Therefore, we speculated that the N protein might have the ability to inhibit dsRNA-activated antiviral activities in a manner similar to that of the VACV E3 protein (21–23). To test this hypothesis, we expressed the N protein in VACVΔE3ΔK3, which can replicate only in cells defective in PKR and OAS/RNase L (18; Cao, unpublished observation). Thus, if SARS-CoV-2 N has the function of inhibiting PKR and RNase L, recombinant VACVΔE3ΔK3 expressing the N protein (VACVΔE3ΔK3/N) would be able to replicate in cells with functional PKR and RNase L (e.g., HeLa and A549 cells), which are not permissive for VACVΔE3ΔK3.

**FIG 2:**
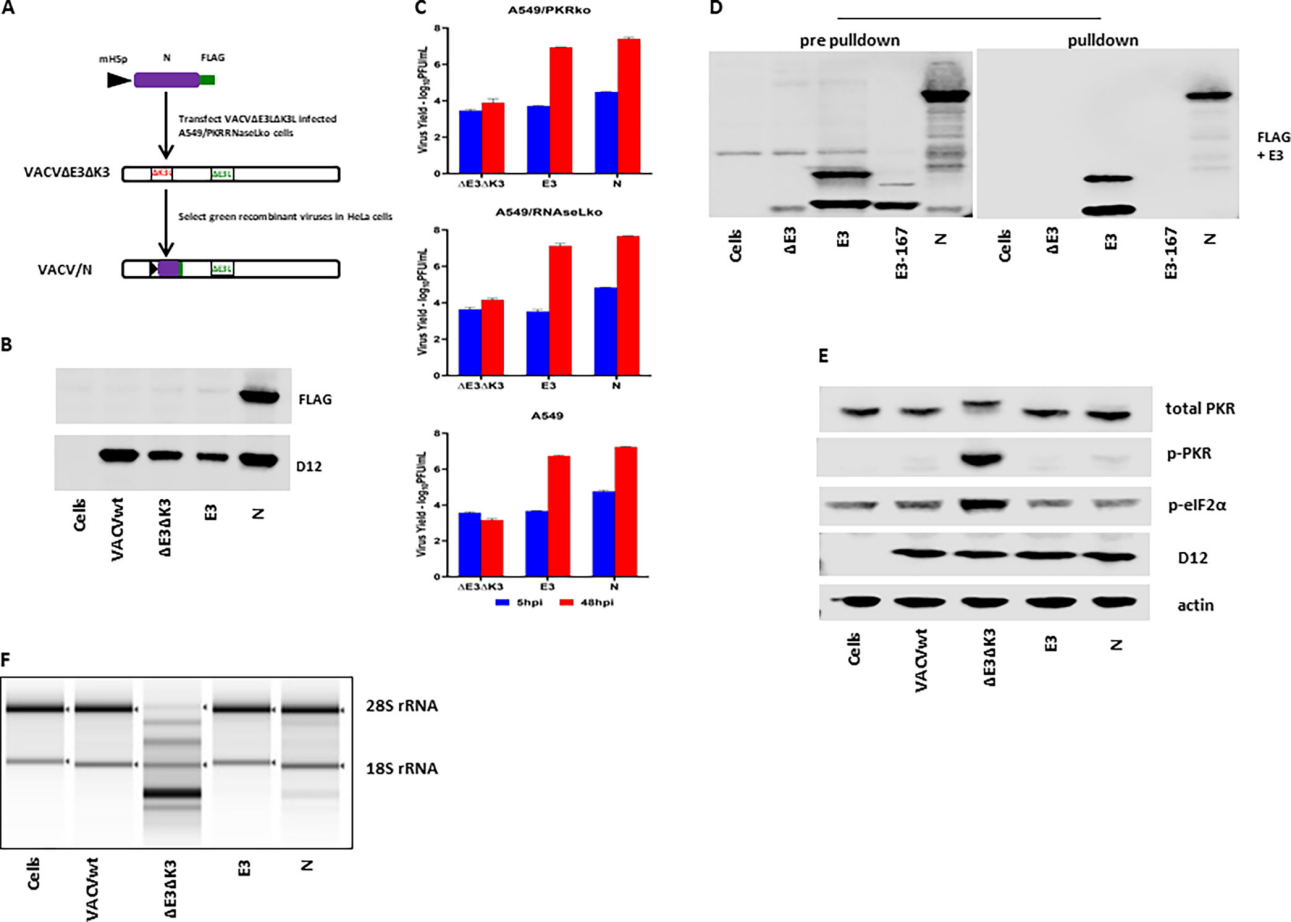
SARS-CoV-2 N binds to dsRNA and inhibits PKR and OAS/RNase L. (A) Schematic illustration of the construction of recombinant VACVΔE3ΔK3 expressing SARS-CoV-2 N. The SARS-CoV-2 N gene tagged at the C terminus with FLAG and driven by the VACV early/late promoter mH5 was inserted into the K3L locus to replace the red fluorescent protein marker of VACVΔE3ΔK3. Infection with VACVΔE3ΔK3 and transfection with the recombinant vector were done in A549/dko cells, and the selection and purification of the recombinant were done in HeLa cells. (B) Confirmation of the expression of SARS-CoV-2 N. Western blot analysis was used to confirm the expression of the SARS-CoV-2 N protein using a FLAG Ab. The cell lysate was prepared using A549/dko cells. D12 is a VACV early protein and was used as an infection control. VACVwt, wild-type VACV; ΔE3ΔK3, VACVΔE3ΔK3; E3, VACVΔK3 expressing VACV E3; N, VACVΔE3ΔK3 expressing SARS-CoV-2 N. (C) Replication of VACVΔE3ΔK3 expressing SARS-CoV-2 N. A549/PKRko, A549/RNaseLko, and wild-type A549 cells were infected with the viruses (shown in panel B) at an MOI of 5. The differences in the virus titers at 48 hpi and 5 hpi indicate the virus yields. The data are representative of results from three independent experiments. (D) dsRNA binding assay by poly(I·C) bead pulldown. BHK21 cells were infected with VACVΔE3 and transfected with the recombinant vectors shown in panel A. E3, VACV E3-expressing vector; E3-167, E3/K167A mutant; N, SARS-CoV-2 N. The protein complex precipitated with poly(I·C) beads was analyzed using Western blotting with FLAG and E3 antibodies. The data are representative of results from three independent experiments. (E) Status of PKR phosphorylation. A549/RNaseLko cells were infected with wild-type VACV (VACVwt), VACVΔE3ΔK3 (ΔE3ΔK3), VACVΔK3 expressing VACV E3 (E3), and VACVΔE3ΔK3 expressing SARS-CoV-2 N (N) at an MOI of 5 for 8 h. The cell lysate was analyzed using Western blotting with Abs for PKR (both phosphorylated and nonphosphorylated), p-PKR (phosphorylated PKR), p-eIF2α (eIF2α phosphorylated at S51), the VACV early/late protein D12, and actin. The data are representative of results from three independent experiments. (F) RNA electrophoresis. A549/PKRko cells were infected with wild-type VACV, VACVΔE3ΔK3, VACVΔK3 expressing VACV E3, and VACVΔE3ΔK3 expressing SARS-CoV-2 N at an MOI of 5. The total RNA collected at 12 hpi was analyzed by gel electrophoresis. The data are representative of results from two independent experiments.

The SARS-CoV-2 N gene driven by a vaccinia virus early/late promoter, mH5 (24), was inserted into VACVΔE3ΔK3, as we described previously (Fig. 2A) (24, 25). As we speculated, recombinant VACVΔE3ΔK3 expressing the N protein can replicate in HeLa cells and thus could be easily selected from the cells. The expression of the N protein by the recombinant viruses was confirmed using a FLAG Ab (Fig. 2B). The replication capacity of VACVΔE3ΔK3/N was further examined and compared to those of VACVΔE3ΔK3 and VACVE3 (expressing E3) in A549 cells (which have functional PKR and RNase L pathways), PKR knockout A549 cells (A549/PKRko), and RNase L knockout A549 cells (A549/RNaseLko) (26). The cells were infected with the viruses at a multiplicity of infection (MOI) of 5, and the virus was collected at 5 and 48 hpi. The virus titers were determined using a plaque assay in A549 PKR/RNase L double-knockout cells (A549/dko), which are permissive for all of the viruses, including VACVΔE3ΔK3 (data not shown). The increase in the virus titer at 48 hpi in comparison to that of the sample collected at 5 hpi indicates the virus yield. As shown in Fig. 2C, VACVΔE3ΔK3/N replicated to a level similar to that of VACVE3 in all three A549 cell lines. In contrast, VACVΔE3ΔK3 was not able to replicate in A549 wild-type cells (A549/wt), and only low virus yields (less than 0.2 logs) were observed in A549/PKRko and A549/RNaseLko cells. Thus, SARS-CoV-2 N can supplement VACV E3 to restore VACVΔE3ΔK3 replication in cells with functional PKR and RNase L pathways.

Next, we examined the dsRNA binding capacity of SARS-CoV-2 N. Previously, we examined VACV E3 and poxvirus E3 orthologs for their dsRNA binding using poly(I·C)-conjugated Sepharose beads (27). Here, wild-type VACV E3 (VACVwt) was used as the positive control, while an E3 mutant, E3/K167A, which was defective in dsRNA binding (28), was used as the negative control. Similar to VACV E3, the SARS-CoV-2 N protein was pulled down with poly(I·C) beads, while the E3/K167A mutant was not (Fig. 2D). Thus, similar to VACV E3, the SARS-CoV-2 N protein is a dsRNA binding protein.

The VACV E3 protein is a potent inhibitor of PKR and OAS/RNase L antiviral activities. When the gene encoding the E3 protein is deleted, the VACV deletion mutant virus induces PKR phosphorylation at threonine 446 and activates OAS/RNase L, as shown by the degradation of rRNAs. As shown in Fig. 2E and F, VACVΔE3ΔK3 induced significant PKR phosphorylation (A549/RNaseLko cells) and degradation of rRNAs (A549/PKRko cells). In contrast, the PKR phosphorylation and RNase L activities in VACVΔE3ΔK3/N-infected cells were similar to those in cells infected with VACV expressing E3 (VACVwt and VACVE3). Thus, the SARS-CoV-2 N protein functions similarly to VACV E3 in inhibiting the activation of the PKR and RNase L pathways.

### The LKR and CTD of SARS-CoV-2 N are the determinants of dsRNA binding and inhibition of PKR/RNase L antiviral pathways.

**FIG 3:**
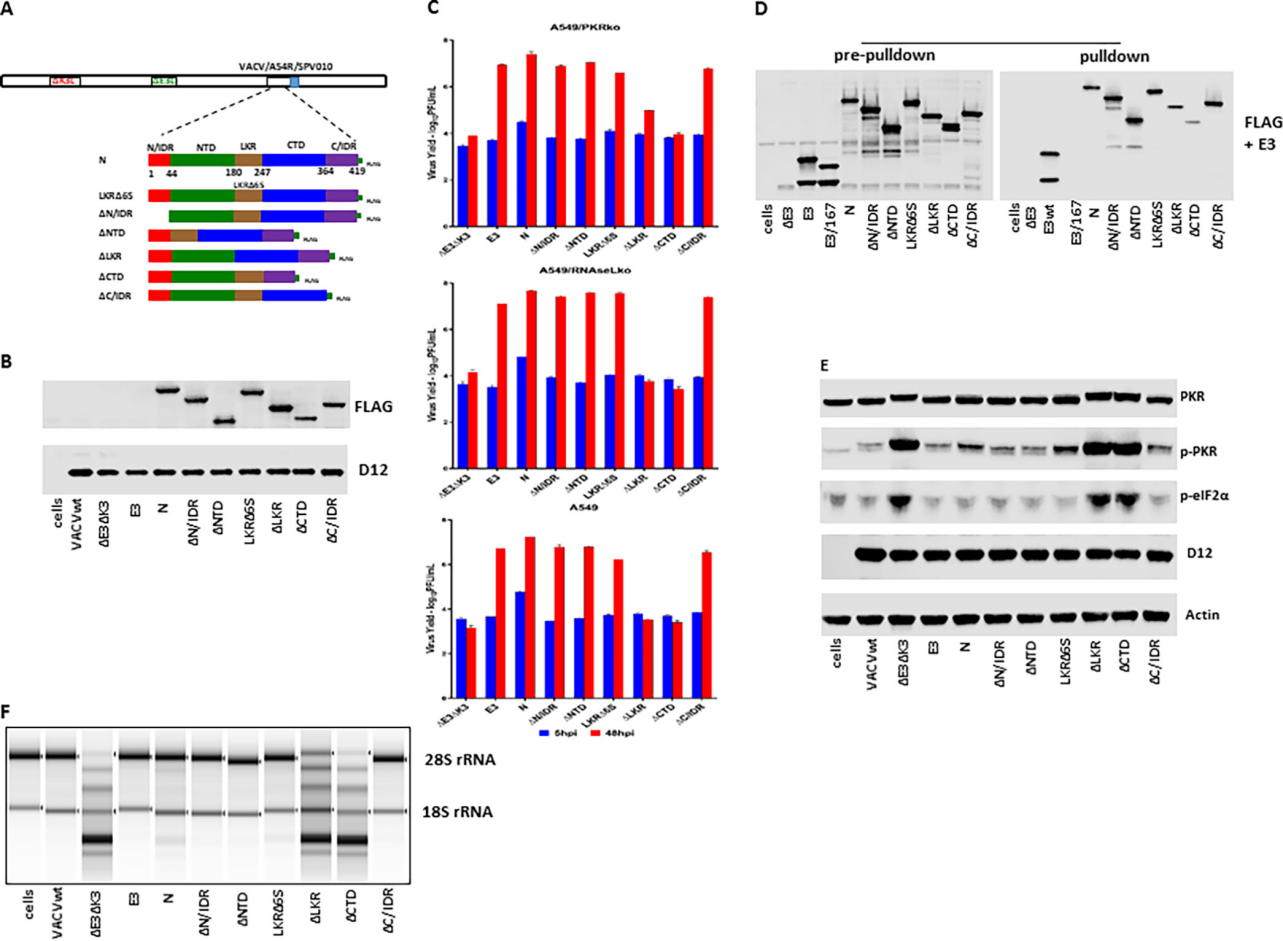
dsRNA binding and inhibition of PKR and OAS/RNase L by SARS-CoV-2 truncated mutants. (A) Schematic illustration of the construction of recombinant VACVΔE3ΔK3 expressing SARS-CoV-2 N truncated mutants. All of the truncated mutants were tagged with FLAG at their C termini and inserted into VACVΔE3ΔK3 at the A45R locus with the swinepox virus K3L ortholog SPV010 as a selection marker. The numbers under full-length N indicate the amino acids. Infection with VACVΔE3ΔK3 and transfection with the recombinant vector were done in A549/dko cells, and the selection and purification of the recombinant were done in PK15 pig cells. (B) Confirmation of the expression of the SARS-CoV-2 N truncated mutants. Western blotting was used to confirm the expression of all truncated N proteins as described in the legend of Fig. 2B. (C) Replication of VACVΔE3ΔK3 expressing the truncated SARS-CoV-2 N mutants. Infection and titration were performed as described in the legend of Fig. 2C. (D) dsRNA binding of the truncated N proteins by poly(I·C) bead pulldown. The assay was performed as described in the legend of Fig. 2D. (E) The status of PKR phosphorylation in cells infected by VACVΔE3ΔK3 expressing the N truncated mutants was examined as described in the legend of Fig. 2E. (F) RNA electrophoresis. The OAS/RNase L activity in cells infected by the N truncated mutants was examined as described in the legend of Fig. 2F.

Coronavirus N proteins have a modular organization consisting of two structural domains, the N-terminal domain (NTD) and the C-terminal domain (CTD), flanked by three intrinsically disordered regions (IDRs), the N-terminal IDR (N/IDR), the linker region (LKR), and the C-terminal IDR (C/IDR) (29, 30). We constructed recombinant VACVΔE3ΔK3 expressing mutants with truncations of each domain of the SARS-CoV-2 N protein and examined their roles in dsRNA binding and the inhibition of PKR and RNase L (Fig. 3). Additionally, we constructed a mutant in which a stretch of 6 serine amino acids (aa) (aa 183 to 190, which also includes 185R and 189R) in the serine-rich motif of the LKR was deleted (LKRΔ6S). The mutant genes, containing a FLAG tag at the C terminus and driven by the vaccinia virus early/late promoter mH5, were inserted into the nonessential A45R gene of VACVΔE3ΔK3 (31). To facilitate the selection of recombinant VACVΔE3ΔK3 expressing the truncated mutants, the swinepox virus K3 ortholog SPV010, which is capable of rescuing the virus only in pig cells (18), was used as a selection marker to select and purify the recombinant viruses in pig cells.

The proteins of all of the truncated SARS-CoV-2 N mutants were expressed from VACVΔE3ΔK3-infected A549/dko cells at comparable levels (Fig. 3A and B). In comparison to the full-length N protein, the deletions of the N/IDR, NTD, LKR6S, and C/IDR (ΔN/IDR, ΔNTD, LKRΔ6S, and ΔC/IDR) had little effect on the ability to rescue VACVΔE3ΔK3 replication in wild-type A549, A549/PKRko, and A549/RNaseLko cells. In contrast, the mutants with a deletion of the LKR or the CTD (ΔLKR and ΔCTD) were not able to rescue VACVΔE3ΔK3 replication in A549 and A549/RNaseLko cells (Fig. 3C), indicating that both the LKR and CTD are required for the inhibition of PKR activities. In contrast, in A549/PKRko cells (which still express RNase L), while the ΔCTD virus was not able to grow, the ΔLKR virus was able to grow with a reduced efficiency (1 log versus 3 logs).

The dsRNA binding capacity and the inhibition of the PKR activation and RNase L activity of the truncated SARS-CoV-2 N mutants were analyzed as described above. As shown in Fig. 3D, the deletion of the N/IDR, NTD, and C/IDR had no effect on the dsRNA binding activity. In contrast, the dsRNA binding of the ΔCTD virus was significantly reduced, while it was slightly reduced by the deletion of LKR (ΔLKR). Correspondingly, the ΔLKR virus inhibited RNA degradation more than the ΔCTD virus in A549/PKRko cells (Fig. 3F). However, PKR was phosphorylated to similar degrees in A549/RNaseLko cells infected with the ΔLKR and ΔCTD viruses (Fig. 3E). These data correlate well with the virus yield data shown in Fig. 3C.

### The LKR and CTD are sufficient for inhibition of PKR and RNase L antiviral activities.

**FIG 4:**
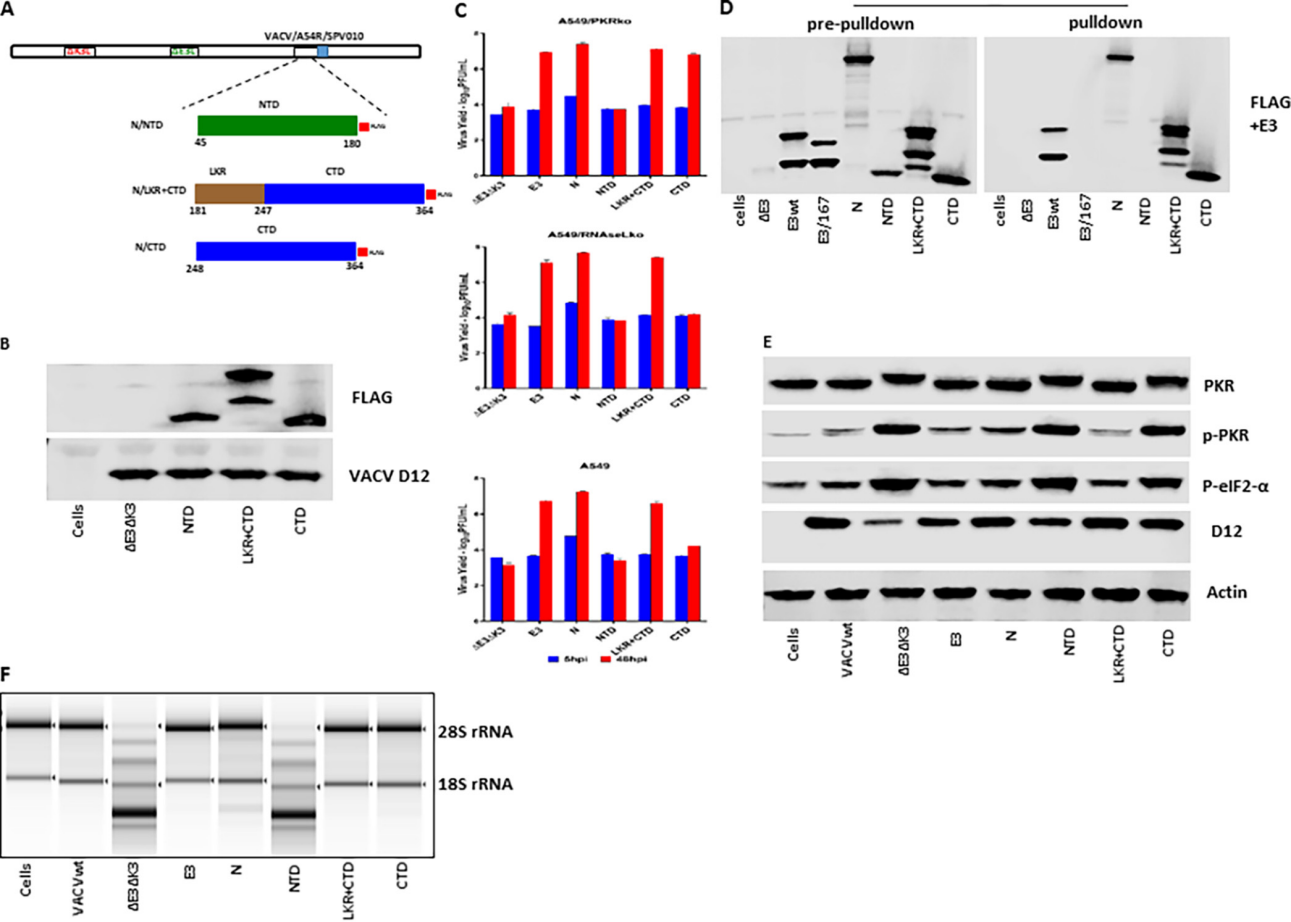
dsRNA binding and inhibition of PKR and OAS/RNase L by SARS-CoV-2 N domain mutants. (A) Schematic illustration of the construction of recombinant VACVΔE3ΔK3 expressing the SARS-CoV-2 N domain mutants. Only the NTD, LKR+CTD, and CTD mutants are shown since no protein was detected for the other domain mutants (C/IDR, LKR, and N/IDR). The construction of the recombinants expressing the N domain mutants was the same as the procedure described in the legend of Fig. 3A. (B) Confirmation of the expression of the SARS-CoV-2 N domain mutants. Western blotting was used to confirm the expression of the N domain mutant proteins as described in the legend of Fig. 2B. (C) Replication of VACVΔE3ΔK3 expressing the SARS-CoV-2 N domain mutants. Infection and titration were done as described in the legend of Fig. 2C. (D) dsRNA binding of the N domain mutant proteins by poly(I·C) bead pulldown. The assay was performed as described in the legend of Fig. 2D. (E) The status of PKR phosphorylation in cells infected by VACVΔE3ΔK3 expressing the N domain mutants was examined as described in the legend of Fig. 2E. (F) RNA electrophoresis. The OAS/RNase L activity in cells infected by the N domain mutants was examined as described in the legend of Fig. 2F.

Next, we examined if the LKR and CTD themselves could perform the function of inhibiting PKR and OAS/RNase L. The individual domains (N/IDR, NTD, LKR, CTD, and C/IDR) and a dual LKR-CTD (LKR+CTD), which were driven by the vaccinia virus early/late promoter mH5, were inserted into VACVΔE3ΔK3 as described above (Fig. 3A). Although transcribed, no protein was detected for the three IDR constructs (N/IDR, LKR, and C/IDR) (data not shown). The protein expression levels of the NTD, LKR+CTD, and CTD constructs are shown in Fig. 4B.

Similar to the full-length N protein, the LKR+CTD was fully functional in rescuing the replication of VACVΔE3ΔK3 in A549/wt, A549/PKRko, and A549/RNaseLko cells (Fig. 4C). Furthermore, the CTD was similar to the full-length N protein and the LKR+CTD in rescuing the replication of VACVΔE3ΔK3 in A549/PKRko cells. However, the virus expressing the CTD showed significantly less replication in A549/wt and almost no replication in A549/RNaseLko cells in comparison to A549/PKRko cells. This indicates that the CTD by itself can inhibit RNase L activity but is not able to inhibit PKR. VACVΔE3ΔK3 expressing the NTD was not able to replicate in A549/wt, A549/PKRko, or A549/RNaseLko cells.

The CTD and the LKR+CTD bound to poly(I·C), while the NTD could not (Fig. 4D). In A549/RNaseLko cells (expressing PKR), the LKR+CTD demonstrated a potency similar to that of the full-length N protein in inhibiting the phosphorylation of PKR and eIF2α, while the CTD and NTD did not (Fig. 4E). The LKR+CTD and the CTD completely inhibited RNase L activity in A549/PKRko cells, while the NTD did not (Fig. 4F).

### Bat-CoV RaTG13 nucleocapsid protein is capable of inhibiting human PKR and RNase L.

**FIG 5:**
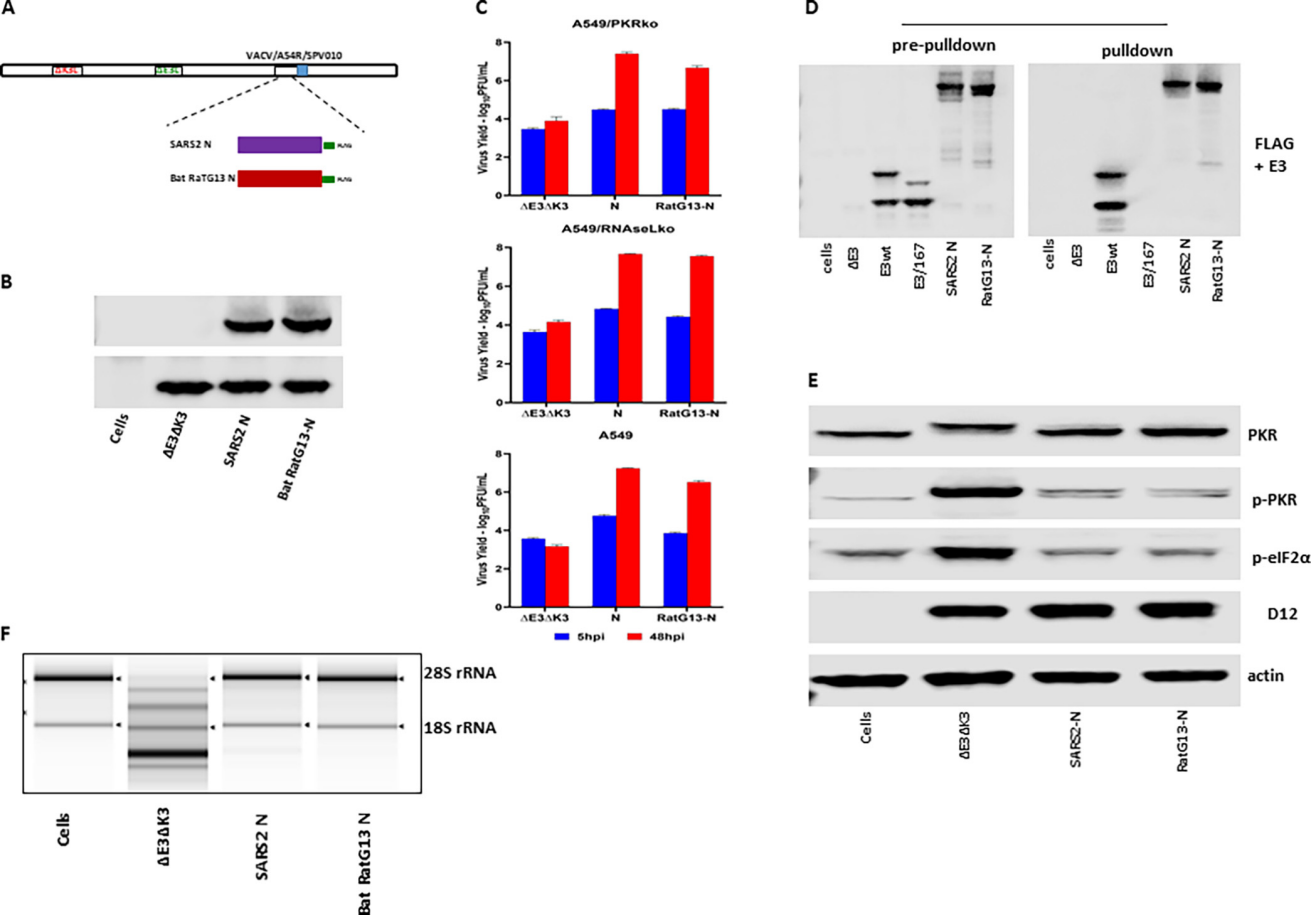
dsRNA binding and inhibition of PKR and OAS/RNase L by bat-CoV RaTG13 N domain mutants. (A) Schematic illustration of the construction of recombinant VACVΔE3ΔK3 expressing the bat-CoV RaTG13 N protein. The bat-CoV RaTG13 N gene tagged with a FLAG tag at the C terminus and driven by the VACV early/late promoter mH5 was inserted into VACVΔE3ΔK3 as shown in Fig. 3A. (B) Confirmation of the expression of the bat-CoV RaTG13 N protein using Western blotting as described in the legend of Fig. 2B. (C) Replication of VACVΔE3ΔK3 expressing the bat-CoV RaTG13 N protein. Infection and titration were done as described in the legend of Fig. 2C. (D) dsRNA binding of the bat-CoV RaTG13 N protein by poly(I·C) bead pulldown. The assay was performed as described in the legend of Fig. 2D. (E) The status of PKR phosphorylation in cells infected by VACVΔE3ΔK3 expressing the bat-CoV RaTG13 N protein was examined as described in the legend of Fig. 2E. (F) RNA electrophoresis. The OAS/RNase L activity in cells infected by bat-CoV RaTG13 N was examined as described in the legend of Fig. 2F.

The closest coronavirus to SARS-CoV-2 is a bat coronavirus (bat-CoV), RaTG13, with 96.2% genome sequence identity (32). For the N protein, there are only 4 amino acid residues (S37P, G215S, G243S, and A267Q) that vary between the two coronaviruses. Interestingly, 3 out of the 4 residues (G215S, G243S, and A267Q) are in the LKR and CTD regions (Fig. 3A), which are important for the function of inhibiting PKR and/or RNase L (Fig. 3 and 4). Here, we mutated these residues in SARS-CoV-2 N to the residues of RaTG13 N. The RaTG13 N protein was expressed in VACVΔE3ΔK3 and analyzed as described above (Fig. 5A and B).

VACVΔE3ΔK3 expressing the bat-CoV RaTG13 N protein replicated at a level comparable to that of VACVΔE3ΔK3 expressing SARS-CoV-2 N in A549/PKRko, A549/RNaseLko, and A549/wt cells, while VACVΔE3ΔK3 did not grow (Fig. 5C). Similar to SARS-CoV-2 N, bat-CoV RaTG13 N bound to poly(I·C) (Fig. 5D) and inhibited PKR phosphorylation and RNase L-mediated RNA degradation (Fig. 5E and F). Thus, the N protein from the bat-CoV is fully capable of inhibiting human PKR- and OAS/RNase L-mediated innate antiviral activities.

### SARS-CoV-2 N specifically binds to phosphorylated PKR.

**FIG 6:**
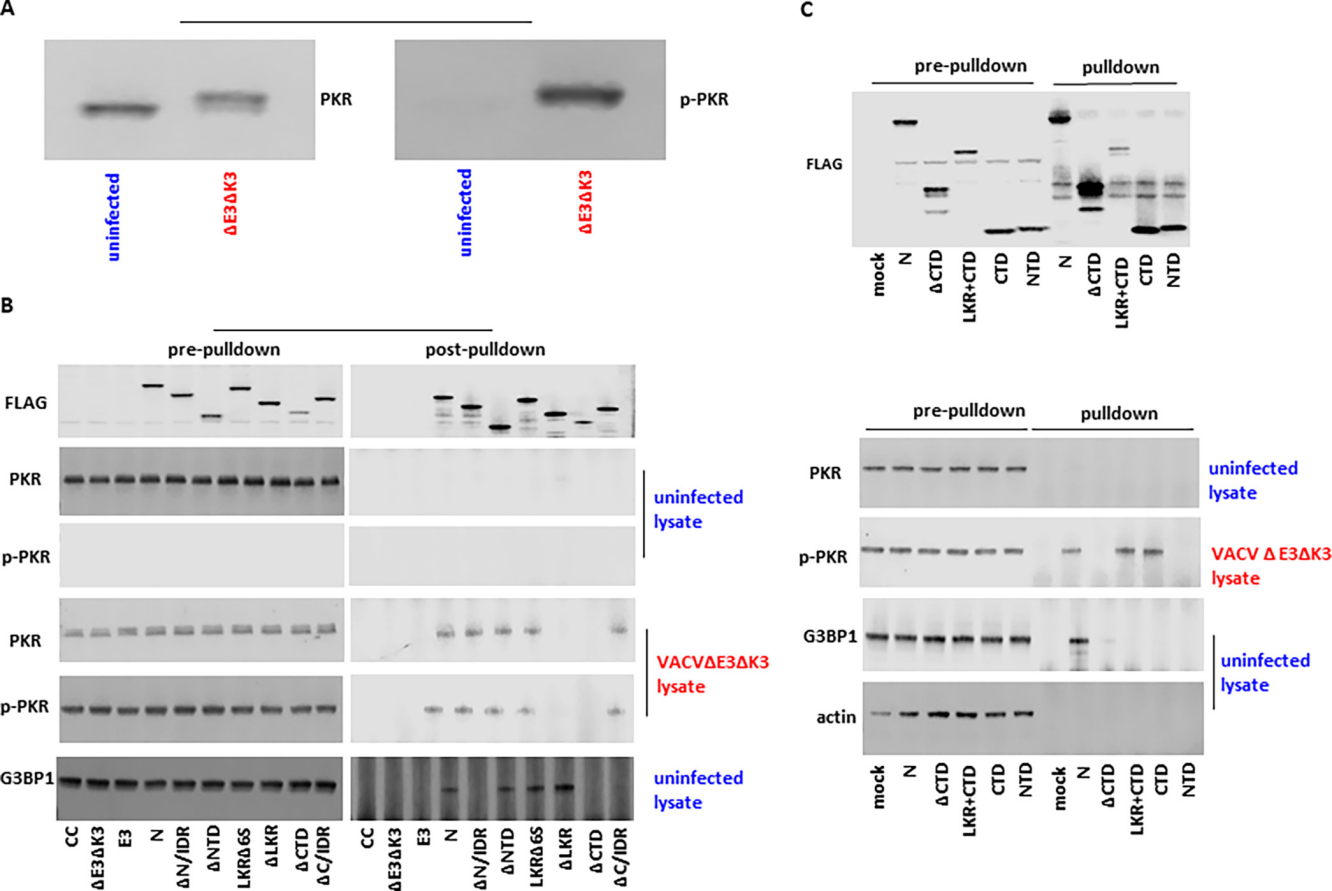
Coimmunoprecipitation of the SARS-CoV-2 N protein and its truncated and domain mutants with PKR and G3BP1. (A) Total PKR and phosphorylated PKR in the cell lysates from uninfected and VACVΔE3ΔK3-infected A549 cells. The cell lysates from uninfected and VACVΔE3ΔK3-infected A549 cells were checked with antibodies for total PKR (left) and PKR phosphorylated at T446 (right). (B) Immunoprecipitation of SARS-CoV N and its truncated mutant proteins. Cell lysates containing SARS-CoV-2 N and its truncated mutant protein were incubated with cell lysates from uninfected or VACVΔE3ΔK3-infected A549 cells (shown in panel A) and precipitated with FLAG antibody and protein G beads. The protein complexes were analyzed using Western blotting with antibodies for the FLAG tag, total PKR, phosphorylated PKR (p-PKR), and G3BP1. The data are representative of results from three independent experiments; CC, cell control. (C) Immunoprecipitation of SARS-CoV N and its domain mutant proteins. The protein complexes containing SARS-CoV N, its domain mutant proteins, and their binding partners were prepared and analyzed as described above for panel B. The data are representative of results from three independent experiments.

A previous study reported that SARS-CoV-2 N could bind to PKR and G3BP1 using an ectopic-overexpression assay (14). Here, we performed a coimmunoprecipitation assay to examine if the SARS-CoV-2 N protein binds to PKR and G3BP1 in the context of virus infection. Since the VACVΔE3ΔK3 recombinant viruses expressing the N protein and the N mutants can induce different levels of phosphorylated PKR, we prepared cell lysates containing nonphosphorylated and phosphorylated forms of PKR separately. Nonphosphorylated PKR was prepared using uninfected A549 cells, and there was little of the phosphorylated form of PKR (Fig. 6A). Phosphorylated PKR was prepared using A549 cells infected with VACVΔE3ΔK3, as almost all of the PKR becomes phosphorylated (Fig. 6A). Concurrently, cell lysates containing SARS-CoV-2 N and its mutants were prepared using A549/dko cells, as all of the N proteins are comparably expressed in these cells. The N protein lysate was then incubated with the cell lysates containing nonphosphorylated or phosphorylated PKR. The immune complex was precipitated using a FLAG antibody to the FLAG tag fused to the C termini of the N protein and its mutants.

As shown in Fig. 6B, the N protein and its truncated mutants were precipitated comparably with the FLAG antibody. No PKR was detected by precipitation from the mixture of N and its mutants with the uninfected cell lysate, as detected by either total PKR- or p-PKR-specific antibodies. In contrast, PKR was precipitated in the lysate of VACVΔE3ΔK3-infected cells incubated with N and its truncated mutants that can rescue VACVΔE3ΔK3 replication in cells with functional PKR and OAS/RNase L (i.e., ΔN/IDR, ΔNTD, LKRΔ6S, and ΔC/IDR) but not in the precipitation complex from the ΔLKR and ΔCTD mutants. Similarly, the LKR+CTD and CTD coprecipitated with PKR from the lysate of VACVΔE3ΔK3-infected cells but not in the lysate of uninfected cells (Fig. 6C). The NTD was not precipitated with PKR after incubation with either cell lysate. Thus, the CTD is sufficient for the interaction between SARS-CoV-2 N and the phosphorylated form of PKR.

Previous studies have shown that SARS-CoV-2 N binds to G3BP1 and that the N/IDR and CTD are required for binding (13). In our experiment, we confirmed the results of that previous report, and additionally, we found that the C/IDR is also required for binding to G3BP1, although it is not required for binding to dsRNA and the inhibition of PKR and OAS/RNase L (Fig. 6B). Interestingly, the NTD, LKR+CTD, and CTD mutants were not capable of binding to G3BP1, although the CTD is essential for binding (Fig. 6B and C).

## DISCUSSION

PKR and OAS/RNase L are major components of innate immunity that inhibit virus replication by shutting off viral protein synthesis (7, 33). Once activated by dsRNA produced during virus replication, activated PKR phosphorylates eIF2α, resulting in the inhibition of protein translation, while activated OAS/RNase L inhibits protein synthesis by degrading virus RNAs. Coronaviruses replicate in the endoplasmic reticulum-associated double-membrane vesicles (20), which may shield dsRNA species from being detected by PRRs during the early stage of virus replication. However, it has been known that dsRNA-activated antiviral pathways, e.g., OAS/RNase L, could be activated as early as 9 h postinfection in the case of murine coronavirus (11). Therefore, we speculated that SARS-CoV-2 may have an antagonist interfering with PKR- and OAS/RNase L-mediated antiviral activities during the early times of infection. Since we observed that the SARS-CoV-2 N protein colocalized with dsRNA during the first 24 h postinfection, we performed an investigation of the potential of the N protein to antagonize dsRNA-activated antiviral pathways, the PKR and OAS/RNase L pathways. We found that although SARS-CoV-2 produced dsRNA during the first 24 h of infection in A549/huACE2 cells, PKR and OAS/RNase L were not activated.

Coronavirus N is a multifunctional protein (29, 30). In addition to its function in packaging the virus genomic RNA into the ribonucleoprotein complex, it plays a role in many aspects of virus replication, such as modulating the cellular environment to facilitate virus replication (13–15, 29, 30, 34, 35). Using an ectopic-overexpression system, the SARS-CoV-2 N protein has been shown to inhibit PKR phosphorylation and the formation of stress granules (SGs) induced by the synthetic dsRNA poly(I·C) (13, 14). However, the antagonizing function of the N protein against PKR and RNase L in the context of virus infection has not been examined. Since the N protein is essential for SARS-CoV-2 replication, we performed the investigation in the context of virus infection using a surrogate VACV system in which the VACV antagonists of the PKR and OAS/RNase L pathways were deleted (VACVΔE3ΔK3). VACVΔE3ΔK3 is a well-established system in which the virus is sensitive to PKR- and OAS/RNase L-mediated antiviral activities and is unable to replicate in cells with functional PKR and/or OAS/RNase L pathways (18; Cao, unpublished observation). The replication capacity of recombinant VACVΔE3ΔK3 expressing SARS-CoV-2 N was comparable to that of VACV expressing the E3 protein in A549, A549/PKRko, and A549/RNaseLko cells. SARS-CoV-2 N inhibited the activation of PKR and OAS/RNase L. Moreover, based on our initial observation that the SARS-CoV-2 N protein colocalized with dsRNA, we further demonstrated that the N protein was capable of binding to dsRNA. Thus, in comparison to previous studies (13, 14), we clearly demonstrate that the SARS-CoV-2 N protein is a dsRNA binding protein that is capable of inhibiting PKR activation and OAS/RNase L activity in the context of virus infection.

Coronavirus N is organized into five distinct domains, including three IDRs (N/IDR, LKR, and C/IDR) and two structural domains (NTD and CTD). Previous studies have shown that the NTD, LKR, and CTD can bind to viral RNA (29, 30). In the previous reports using the ectopic overexpression of truncated mutants of the N protein, the LKR and the CTD plus the C/IDR were shown to be critical for inhibiting the formation of SGs and PKR activation by poly(I·C). In this study, we found that the CTD is responsible for dsRNA binding, while the NTD and LKR are dispensable. Interestingly, the CTD itself is capable of binding to dsRNA and inhibiting OAS/RNase L. However, both the LKR and CTD are required for the complete inhibition of PKR activation as demonstrated by the truncated mutants and individual domains of the N protein (Fig. 3 and 4). Thus, dsRNA binding capability is essential, but not sufficient, for the function of the N protein in inhibiting PKR. Further studies are required to reveal the mechanisms of how the N protein inhibits PKR antiviral activities. Liquid-liquid-phase separation (LLPS) has recently been shown to be an important mechanism for organizing a wide range of biological processes of cells, and there has been increasing evidence that LLPS plays a critical role in viral replication and the modulation of host-cell interactions (36, 37). Numerous studies have demonstrated the association between LLPS of SARS-CoV-2 N and its functions in packaging viral RNA and modulating cellular innate immune responses, e.g., the formation of SGs (15, 38–45). In previous studies on the LLPS of SARS-CoV-2 N, various types of RNA were used to drive phase separation. Closely related to what we found in this report is a biophysical analysis of SARS-CoV-2 N-mediated LLPS where the CTD termed RNA binding domain 2 (RBD2) is responsible for the interaction with dsRNA that drives phase separation, while the NTD (RBD1) is not required (46). Future studies to investigate the functional association between the dsRNA-driven LLPS of SARS-CoV-2 N and the inhibition of PKR and OAS/RNase L should aid in understanding the mechanism that the N protein uses to modulate these antiviral activities.

It has been reported that SARS-CoV-2 N can physically interact with PKR and G3BP1 to inhibit SG assembly using an ectopic-expression system (13, 14). We performed a coimmunoprecipitation assay to examine the physical interaction between SARS-CoV-2 N and PKR/G3BP1 in the context of virus infection. Most interestingly, we found that the N protein and its mutants (truncated mutants and domain mutants) are capable of inhibiting PKR activation and specifically binding to the phosphorylated form of PKR. The previous report showing binding between the N protein and PKR used a FLAG-tagged PKR expression system and could not distinguish between the two forms of PKR (14). Our observation suggests that in addition to the inhibition of PKR activation, SARS-CoV-2 N may also interfere with the function of activated PKR. To our knowledge, this is the first observation that a viral protein specifically binds to the activated form of PKR. Further studies are required to validate the functional consequence of this specific binding of phosphorylated PKR and if it is linked to dsRNA binding. In this investigation, we confirmed previous reports that SARS-CoV-2 N binds to G3BP1, a key component of SGs in the context of virus infection (13, 14). However, the truncated N mutants that are capable of inhibiting PKR and OAS/RNase L, such as ΔN/IDR and ΔC/IDR, lost their G3BP1 binding ability. Similarly, the N domain mutant LKR+CTD, although capable of inhibiting PKR and OAS/RNase L, did not bind to G3BP1. Thus, although our data revealed that G3BP1 binding requires the N/IDR, CTD, and C/IDR, binding to G3BP1 by the N protein is not related to the inhibition of PKR and OAS/RNase L.

Based on epidemiological and genomic sequence data, it is generally accepted that the origin of SARS-CoV-2 was a zoonotic spillover from an animal source, although the exact animal reservoir for the virus has not been identified (4, 47). The closest virus to SARS-CoV-2 is a bat coronavirus, RaTG13, which shares over 90% sequence identity with all open reading frames (ORFs) of SARS-CoV-2 (32). The spike protein of bat-CoV RaTG13 can bind to human ACE2 and infect human cells (48, 49). The N protein of bat-CoV RaTG13 shares over 99% amino acid identity with SARS-CoV-2. In this study, we demonstrate that the N protein of the bat-CoV is capable of inhibiting human innate antiviral immunity mediated by PKR and OAS/RNase L, thus further illustrating that the bat-CoV RaTG13 has the potential to cause infection in humans. However, it should be noted that the ability of the SARS-CoV-2 and bat-CoV N proteins to inhibit dsRNA-activated antiviral activities needs to be investigated in the context of SARS-CoV-2/bat-CoV infection in human cells.

It is known that coronaviruses encode proteins to inhibit dsRNA-activated pathways. Some coronaviruses encode virus-specific proteins to inhibit dsRNA-activated antiviral pathways. For example, the murine coronavirus nonstructural protein ns2 and MERS-CoV ns4b can cleave 2′,5′-oligoadenylate and inhibit the OAS/RNase L pathway (11, 50). The MERS-CoV ns4a protein is a dsRNA binding protein and is capable of inhibiting the PKR-mediated formation of SGs induced by plasmid transfection (51, 52). However, the deletion of ns4a from MERS-CoV does not lead to a significant activation of the PKR and OAS/RNase L pathways (50). Interestingly, counterpart proteins of MHV ns2 and MERS-CoV ns4a and -4b are not present in SARS-CoV-2. A nonstructural protein, nsp15 (endoribonuclease), conserved among coronaviruses, including SARS-CoV-2, has been shown to inhibit dsRNA accumulation, PKR activation, and the formation of stress granules (53, 54). In the case of MERS-CoV, however, only the combinational deletion of ns15 and ns4a or -4b could abolish the inhibition of dsRNA-induced antiviral pathways, while the single inactivation of ns15 could not (55). In this report, we demonstrate that the highly conserved N protein is capable of inhibiting both the PKR and OAS/RNase L pathways in a surrogate virus system. It would be interesting to further investigate if the N protein or the N protein in combination with ns15 would function as an antagonist of the dsRNA-induced antiviral pathways during SARS-CoV-2 infection. Using a similar VACV surrogate system, it was shown previously that the N protein of MHV A59 could partially inhibit OAS/RNase L but not PKR activation in human cells (56). Therefore, it is likely that N proteins of different coronaviruses may have different capabilities of inhibiting dsRNA-induced antiviral pathways, although the N protein is highly conserved among coronaviruses.

The coronavirus N protein is the most abundant and highly antigenic structural protein (57). Several COVID-19 vaccine candidates based on the application of both spike and N as antigens have been shown to be protective against SARS-CoV-2 challenge in animal models such as hamsters and nonhuman primates (NHPs) (58–60). Here, we found that N is a dsRNA binding protein capable of inhibiting PKR- and OAS/RNase L-mediated antiviral activities. In addition, previous studies have shown that the N protein has the capacity to inhibit IFN expression (35). Therefore, for application as an antigen in vaccine development, an N mutant that keeps its antigenic integrity but without inhibiting host innate immune responses should be investigated.

In this study, we demonstrate that SARS-CoV-2 N has the capacity to inhibit two important innate antiviral pathways, PKR and OAS/RNase L. It is logical that SARS-CoV-2 N plays an important role in the high transmissibility and pathogenicity of SARS-CoV-2, which are two viral determinants of the COVID-19 pandemic. Based on our findings that the LKR and CTD of the N protein are required to inhibit PKR and OAS/RNase L activities, we are investigating the domain/motif of the N protein that is essential only for PKR and OAS/RNase L inhibition but not for virus replication. This will allow us to analyze the immune-modulating function of the N protein in the context of SARS-CoV-2 infection. The findings in this study are valuable for designing novel antivirals targeting the N protein and for modifying the N protein as a protective antigen for the development of N protein-based vaccines.

## MATERIALS AND METHODS

### Cells and viruses.

A549, A549 PKR knockout (A549/PKRko), A549 RNase L knockout (A549/RNaseLko), and A549 PKR/RNase L double-knockout (A549/dko) cells were provided by B. Moss (NIH, USA) (26). A549 cells expressing human ACE2 and TMPRSS2 were purchased from InvivoGen (San Diego, CA, USA). PK15, BHK21, and HeLa cells were obtained from the ATCC. All cells were cultured in Dulbecco’s modified Eagle’s medium (DMEM) (Gibco) supplemented with 10% fetal bovine serum (FBS), glutamine, and penicillin-streptomycin at 37°C with 5% CO_2_. The vaccinia virus (VACV) Western Reserve (WR) strain and the E3L and K3L double-deletion mutant (VACVΔE3ΔK3) were described previously (18), and SARS-CoV-2 was an ancestral Canadian isolate (hCoV/Canada/ON-VIDO-01/2020) described previously (61). The VACV-related experiments were performed in our biosafety level 2 (BSL-2) laboratory, and SARS-CoV-2 infections were done in our BSL-3/4 containment laboratory.

### Construction of recombinant VACV.

VACVΔE3ΔK3, described previously (18), was used as the parental virus to construct recombinant viruses expressing SARS-CoV-2 nucleocapsid (N) and N mutants. The N and N mutant sequences were based on the SARS-CoV-2 Wuhan isolate (GenBank accession number MN908947). The modular structure of the SARS-CoV-2 N protein was based on an alignment with a SARS virus: the N-terminal intrinsically disordered region (N/IDR) (amino acids 1 to 44), the N-terminal domain (NTD) (amino acids 45 to 180), the linker region (LKR) (amino acids 181 to 247), the C-terminal domain (CTD) (amino acids 248 to 364), and the C-terminal IDR (C/IDR) (amino acids 365 to 419) (29, 30). The full-length N protein, the N truncated mutants (ΔN/IDR, ΔNTD, ΔLKR, ΔCTD, and ΔC/IDR), and the domain mutants (N/IDR, NTD, LKR, CTD, and C/IDR) with a FLAG tag added to their C termini and driven by a VACV early/late promoter, mH5 (24), were synthesized by GenScript (NJ, USA). Full-length N was inserted into the K3L locus of VACVΔE3ΔK3 using a method that we described previously (25). The N mutants were inserted into the A45R locus of VACVΔE3ΔK3, which is nonessential for VACV both *in vivo* and *in vitro* (31), as we previously described (18). Since we expected that some of the N mutants would lose the function of rescuing the replication of VACVΔE3ΔK3 in human cells, the swinepox virus K3L ortholog SPV010, which is capable of rescuing the replication of VACVΔE3ΔK3 only in swine cells, was used as a selection marker to select and purify recombinant VACVΔE3ΔK3 in a pig cell line, PK15 (18). The working stocks of all of the VACVΔE3ΔK3 recombinants expressing N and the N mutants were prepared in A549/dko cells.

### Confocal microscopy.

Cell monolayers in a four-chambered slide (Thermo Fisher) were infected with the virus at a multiplicity of infection (MOI) of 0.5 and incubated at 37°C. At 24 h postinfection (hpi), cells were washed twice with phosphate-buffered saline (PBS) and fixed using a 3% paraformaldehyde–PBS solution for 10 min at room temperature. The fixed cells were washed 3 times with PBS for 3 min and then permeabilized using a 0.2% Triton X-100–PBS solution for 5 min at room temperature, with gentle rotation. Following another 3 washes with PBS (1 min each wash), the cells were incubated with a blocking buffer (2% FBS and 2% bovine serum albumin [BSA] in PBS) at 4°C for 1 h. The cells were then incubated with the primary antibody (SARS-CoV-2 N Ab from Sino Biological or dsRNA J2 Ab from Scicons), diluted 1/200 in blocking buffer, at 4°C for 2 h. The cells were washed 3 times with PBS for 3 min each wash and incubated with a fluorescence-conjugated secondary antibody specific for the primary antibody (Alexa Fluor secondary antibodies; Thermo Fisher), diluted 1/1,000 in blocking buffer, at 4°C for 1 h. The cells were washed 3 more times with PBS for 3 min each. The chambers were removed from the slides and rinsed with water. Prolong Diamond antifade mountant with 4′,6-diamidino-2-phenylindole (DAPI) (Invitrogen) was added, and the slides were covered with a coverslip and incubated at room temperature in the dark overnight. Slides were imaged using a confocal microscope (Observer.Z1; Zeiss) at a ×40 magnification.

### RNA electrophoresis.

Cell monolayers were infected with the viruses listed in the related figure legends at an MOI of 5 and incubated at 37°C for 12 h (VACV) or 24 or 48 h (SARS-CoV-2) after infection. The total RNA was extracted using the RNeasy minikit (Qiagen) according to the manufacturer’s instructions. RNA concentrations were determined using the Qubit 4 system (Thermo Fisher) and diluted to a final concentration of 5 ng/μL. Ten nanograms of the RNA samples was analyzed by RNA electrophoresis using the high-sensitivity RNA ScreenTape system (Agilent) according to the manufacturer’s instructions, and rRNA was visualized using the 2200 TapeStation system (Agilent).

### Poly(I·C) pulldown.

Poly(I·C) (Sigma-Aldrich)-conjugated Sepharose beads (GE Healthcare) were prepared as previously described (28). BHK21 cells were infected with VACVΔE3 at an MOI of 1 and transfected with the recombinant plasmids constructed as described above using Attractene transfection reagent (Qiagen) according to the manufacturer’s protocol. Twenty-four hours after infection and transfection, the cells were washed twice with cold PBS and then lysed using a nondenaturing lysis buffer (20 mM Tris-HCl, 137 mM NaCl, 10% glycerol, 1% Triton X-100, 2 mM EDTA, and a Roche protease inhibitor cocktail) plus 1 round of freezing and thawing. Cell debris was removed by centrifugation at 5,400 × *g* for 10 min, and an aliquot was removed and used as a prepulldown control. The rest of the supernatant was incubated with the poly(I·C)-conjugated Sepharose beads at room temperature for 2 h with end-over-end rotation. The beads were washed 3 times with nondenaturing lysis buffer to remove unbound proteins. Bound proteins were released from the poly(I·C) beads using an SDS-PAGE loading buffer (50 mM Tris-HCl, 2% SDS, 0.1% bromophenol blue, 10% glycerol, and 100 mM 2-mercaptoethanol) with boiling at 95°C for 10 min. Samples were analyzed using Western blotting as described previously (18).

### Immunoprecipitation.

For the preparation of the SARS-CoV-2 N and mutant protein cell lysates, A549/dko cells were infected with the corresponding viruses for 8 h. Following one wash with PBS, the cell lysate was collected using the nondenaturing lysis buffer described above, and nonsoluble cell debris was removed by centrifugation at 2,000 × *g* at 4°C for 5 min. To prepare the cell lysate containing nonphosphorylated or phosphorylated PKR, wild-type A549 cells were mock infected (for nonphosphorylated PKR) or infected with VACVΔE3ΔK3 (for phosphorylated PKR) for 8 h, and the cell lysate was then prepared as described above for the N and mutant cell lysates. Coimmunoprecipitation was performed using the Dynabeads-protein G immunoprecipitation kit (Invitrogen) according to the manufacturer’s instructions. A mouse anti-FLAG antibody (Sigma-Aldrich) was used to bind to SARS-CoV-2 N and its mutants. The final precipitated protein complex was eluted from the Dynabeads-protein G using SDS sample buffer as described above and analyzed using Western blotting as described previously (18).

### Western blotting.

Confluent cell monolayers in a 12-well tissue culture plate were infected with the viruses and collected at the time points indicated in the related figure legends. SDS-PAGE, membrane transfer, antibody blotting, and protein band detection were performed as described previously (18). Total PKR, p-PKR, p-EIF2α, and actin antibodies were purchased from Abcam. FLAG tag antibodies were purchased from Sigma-Aldrich. VACV D12 protein antibody was described previously (27). SARS-CoV-2 N protein antibody was purchased from Sino Biological.
